# Patient Plasma–Based Immunoproteomics Reveals Novel *Echinococcus granulosus* Antigens for Diagnosis of Cystic Echinococcosis

**DOI:** 10.1016/j.mcpro.2025.101485

**Published:** 2025-12-08

**Authors:** Congmin Zhang, Quzhen Gongsang, Wangmu Danzeng, Xi Gao, Yuxin Li, Yanping Zhao, Hongkai Xu, Cong Wang, Ting Zhang, Muxin Chen, Yijun Tang, Jiawei Liu, Jin Zi, Liang Lin, Guixue Hou, Siqi Liu

**Affiliations:** 1 BGI Genomics, Shenzhen, China; 2 NHC Key Laboratory of Echinococcosis Prevention and Control, Tibet Center for Disease Control and Prevention, Lhasa, China; 3 BGI Research, Chongqing, China; 4 National Institute of Parasitic Diseases, Chinese Center for Disease Control and Prevention, Chinese Center for Tropical Diseases Research, Shanghai, China; 5 Shenzhen Center for Disease Control and Prevention, National Institute of Parasitic Diseases, Shenzhen, Guangdong, China

**Keywords:** antigen, biomarker, diagnosis, immunoproteomics, parasite

## Abstract

Cystic echinococcosis (CE), a parasitic disease caused by *Echinococcus granulosus* (Eg), remains prevalent in underdeveloped pastoral regions. Current diagnostic methods for CE primarily rely on imaging techniques, whereas serological tests still require significant improvement. To address this challenge, we have developed an immunoproteomics workflow to identify novel diagnostic Eg antigens. Our approach involved extracting proteins from CE surgical tissues, which were then recognized by patient plasma through immunoblotting and subsequently identified using mass spectrometry. Applying stringent criteria to evaluate Eg protein antigenicity, we selected 25 candidates for expression, and 18 recombinant proteins demonstrated enhanced immunoreactivity with CE patient plasma. Further validation led to the identification of a novel panel comprising eight Eg recombinant antigens, which exhibited superior diagnostic capabilities with sensitivities ranging from 91.26% to 99.09% and specificities ranging from 95% to 97%. This panel was tested in a large-scale study involving 1068 plasma samples collected from patients with ultrasound-confirmed CE (+) and healthy controls. Our findings introduce a set of novel Eg antigens with significant potential for improving CE clinical diagnosis, particularly in its early stages. This research not only advances our understanding of CE immunology but also offers promising tools for enhancing disease detection and management in affected populations.

## Highlights

• Novel immunoproteomics workflow identifies promising *Echinococcus granulosus* antigens.
• Panel of eight recombinant *Echinococcus granulosus* antigens shows enhanced specificity and sensitivity for cystic echinococcosis diagnosis.
• Large-scale validation (n = 1068) demonstrates potential for improved early stage cystic echinococcosis detection.

## Graphical Abstract

**Figure:**
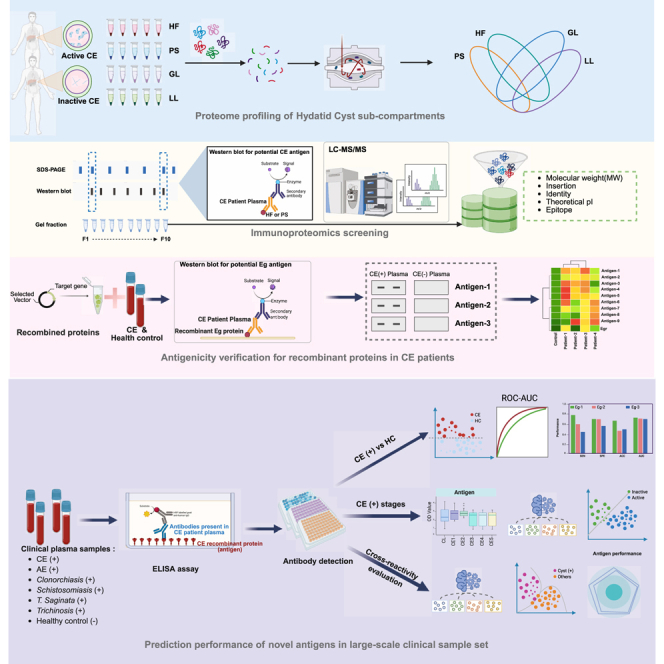

## In Brief

Current diagnostic methods for cystic echinococcosis screening primarily rely on imaging techniques, whereas serological tests still require significant improvement. To address this challenge, we have developed an immunoproteomics workflow to identify eight novel diagnostic *Echinococcus granulosus* antigens. Further validation led to the identification of a novel panel comprising eight *E. granulosus* recombinant antigens, which exhibited superior diagnostic capabilities with sensitivities ranging from 91.26% to 99.09% and specificities from 95% to 97%.

Cystic echinococcosis (CE) is a neglected parasitic disease caused by *Echinococcus granulosus* (Eg), with high incidence in underdeveloped pastoral regions, posing a significant public health threat (1, 2, 3). Current diagnosis relies heavily on ultrasonography, which favors detection of advanced disease but often misses early term and midterm infections (4). Hydatid cysts (HCs) in CE patients exhibit slow growth with minimal size changes over 10 years, making small HCs undetectable by conventional ultrasonography (5). Although the MRI performs excellently for anatomical staging, a single examination costs 5 to 10 times more than a full serological panel, which is prohibitive for large-scale screening in endemic regions (4). While PCR could differentiate between species and genotypes with less invasive samples, it shows low blood-circulating cell-free DNA sensitivity (about 20–25%) and performs poorly with low-parasite–load samples (6). Due to the nonspecific clinical presentation of early stage CE, serological testing serves as a prescreening method (7). The Eg oncosphere provokes innate and acquired immune responses (8), whereas the HC structure facilitates Eg antigen release into circulation, eliciting corresponding immune responses (9). Antibodies against these responses are anticipated to serve as early stage serological indicators for CE (10), making identification of highly immunogenic Eg proteins crucial for improving serodiagnosis.

Despite 30 years of efforts, few Eg antigens, mainly from protoscoleces (PS) and hydatid fluid (HF), have proven clinically suitable (11, 12). Native HF proteins remain the primary source for CE serology testing, but their production poses challenges in specificity, sensitivity, and quality control (QC) (13, 14). Recombinant HF proteins, including antigen B (15), antigen 5 (16), Eg actin filament fragmenting protein (17), calcium-binding protein (18), and thioredoxin peroxidase (19), show better performance compared with native proteins (20). Synthetic peptides derived from HF proteins have not significantly improved sensitivity and specificity (21).

The elusiveness of highly antigenic Eg proteins remains challenging. Antigen research has focused on optimizing antigenicity of specific amino acid fragments from antigen B, antigen 5, and a few other proteins (22). Despite significant proteomics progress over 20 years, the comprehensive identification of antigenic Eg proteins reactive to CE patient sera has been slow. Proteomic studies on Eg remain limited, with fewer than 20 publications identifying a maximum of 280 proteins (23, 24), significantly lower than approximately 5000 proteins annotated in the Eg genome. The differential antigenic potential across HC structures (25) remains underexplored. The two-layered, compact cyst nature may hinder Eg DNA and protein release into HF, whereas some HF proteins demonstrate antigenic properties. Detailed assessment of proteins across all cyst components is essential.

Herein, we present a patient plasma–based immunoproteomics approach to identify and validate antigenic Eg proteins through four phases: (1) integration of immunoassays with mass spectrometry (MS) to identify Eg proteins from diverse HC anatomical structures and CE patient plasma; (2) selection of Eg proteins with predicted antigenic properties for recombinant production; (3) evaluation of recombinant antigens using multiple CE patient plasma samples; and (4) verification of potential biomarkers *via* ELISA, distinguishing healthy individuals from ultrasound-confirmed CE patients, classifying CE stages, and evaluating crossreactivity with other parasitic infections. Our patient plasma–based immunoproteomics approach has the potential to uncover clinically applicable biomarkers, with discovered immunoreactive protein antigens serving as targets for CE diagnostic and treatment management.

## Experimental Procedures

### Clinical Sample Collection

HC tissues were obtained from 10 surgical CE patients at the Second People's Hospital of Tibet Autonomous Region. Plasma samples were collected through collaboration with the Tibet Center for Disease Control and Prevention. The human studies reported in our article abide by the Declaration of Helsinki principles. Ethics approval covering all study samples was granted by the Hospital Institutional Review Board and the Chinese Center for Disease Control and Prevention (approval no.: 2019005 for echinococcosis patients and healthy endemic controls) and the Ethical Review Committee of the National Institute of Parasitic Diseases, Chinese Center for Disease Control (approval no.: 2021019 for cross-reactivity testing patients). Patient classification followed the World Health Organization-Informal Working Group on Echinococcosis criteria (4). For cross-reactivity testing, plasma samples from patients infected with *Clonorchiasis* (N = 16), *Schistosomiasis* (N = 12), *Taenia saginata* (N = 20), *Trichinosis* (N = 13), and *Echinococcus multilocularis* (Em; N = 61) were collected. Blood samples were centrifuged to isolate plasma and stored at −80 °C.

### Protein Extraction and Identification

HC tissues were dissected into four subtissues: PS, HF, germinative layer (GL), and laminated layer (LL). Tissues were homogenized in lysis buffer (7 M urea, 2 M thiourea, 20 mM Tris–HCl, pH 7.4), sonicated, and centrifuged at 20,000*g* for 30 min. For LC–MS/MS analysis, proteins were reduced with 10 mM DTT, alkylated with 100 mM iodoacetamide, and digested with trypsin (1:50 ratio) overnight at 37 °C in 50 mM NH_4_HCO_3_. Peptides were purified using Sep-Pak C18 cartridges. For gel-based analysis, SDS-PAGE bands were destained, reduced with DTT, alkylated with iodoacetamide, and digested with trypsin overnight. Peptides were extracted and dried before LC–MS/MS analysis.

Peptides were analyzed using Ultimate 3000 nano HPLC coupled with a Q Exactive HF mass spectrometer. MS1 resolution was 70,000 (350–1600 *m/z*, automatic gain control = 3E6, and injection time = 100 ms); MS2 resolution was 17,500 (automatic gain control = 50,000, injection time = 120 ms, and normalized collision energy = 27%). The MS raw data were processed with MaxQuant (26) (version 1.6.1.25) against *Eg* UniProt protein database (2023_05_04, including 23,260 entries) and the Swiss-Prot database for *Homo sapiens* (release 2023-02 with 20,598 entries). Search parameters included 10 ppm for MS1 and 0.05 Da for MS2 mass tolerance, carbamidomethylation (C) as fixed modification, deamination and oxidation as variable modifications, and with a maximum of two missed cleavages. Peptides and proteins were filtered at 1% false discovery rate.

### Experimental Design and Statistical Rationale for the MS-Based Proteomics

Proteomic discovery was done in 10 surgically resected HCs, including five active and five inactive CE patients. All the surgery samples were individually dissected into four anatomically distinct subtissues (PS, HF, GL, and LL). All the dissected subtissues underwent protein extraction followed by SDS-PAGE and immunoblotting. Five active-stage CE patient plasma (biological replicates) were treated in the immunoproteomic analysis, and one healthy donor plasma pool was treated as a negative control. The immunoreactive bands were excised and in-gel digested, and the tryptic digestion products were delivered to LC–MS/MS with one technical replicate per band. A detailed experimental design is illustrated in the Graphical Abstract.

### Immunoblotting Analysis

Proteins from HF and PS were separated by SDS-PAGE and transferred to membranes. Membranes were incubated with CE patient plasma (3 h, room temperature), followed by horseradish peroxidase–labeled goat anti-human immunoglobulin G (IgG) (1:250 dilution). Chemiluminescence was detected using the Enhanced Chemiluminescence kit and Amersham Imager 600. Commercial Egr antigen (Hangzhou Immuno Biotech) served as a reference. Band densitometry was quantified using ImageJ software (27).

### Recombinant Protein Expression

Selected Eg proteins were cloned into the pET-31b (+) vector and expressed in *Echinococcus coli* BL21(DE3). Cultures were grown in LB medium with ampicillin (100 μg/ml) at 37 °C. Protein expression was induced with 1 mM IPTG at an absorbance of 0.6 at 600 nm for 6 h. Cells were harvested, lysed by sonication, and His-tagged proteins were purified using nickel–nitrilotriacetic acid agarose under denaturing conditions (8 M urea). Proteins were eluted with buffer containing 300 mM imidazole and stored at −20 °C. Purified proteins were diluted with PBS, with a final urea concentration of <0.5 M, before ELISA coating. A pilot ELISA using blank solvent demonstrated that coating under these conditions gave no significant change of absorbance values compared with direct coating of PBS, confirming that residual urea does not interfere with antigen binding or antibody recognition.

### ELISA Validation

Antigen concentration and plasma dilution were optimized by checkerboard titration. Signal-to-noise ratios determined optimal conditions for each antigen. ELISA was performed in triplicate using 96-well plates coated with recombinant antigens overnight at 4 °C. Wells were blocked with 5% nonfat milk in PBS with Tween-20 for 2 h, incubated with diluted plasma samples (1 h), followed by horseradish peroxidase–conjugated anti-human IgG (1 h). Tetramethylbenzidine substrate was added for 10 min, reactions were stopped with 1 N sulfuric acid, and absorbance was measured at 450 nm. The pooled CE plasma used for plate-to-plate normalization was prepared from randomly selected 100 CE-positive donors in the whole cohort. Once the pool was aliquoted, it was stored at −80 °C until use. Absorbance values were normalized using pooled CE plasma as system reference material. Relative absorbance values were calculated by comparing each sample to the reference standard for cross-plate comparability.

### Statistical Analysis

Statistical analysis was performed using R (version 4.1.3) with ggplot2 for visualization and pROC (28) for receiver operating characteristic analysis. Machine learning models (LASSO, random forest, and support vector machine [SVM]) were trained using fivefold crossvalidation with grid search hyperparameter tuning through the Caret package (29). Performance metrics included accuracy, sensitivity, specificity, and area under the receiver operating characteristic curve (AUROC).

## Results

### Immunoproteomic Profiling of Eg Proteins Across HC Subtissues

**Fig. 1:**
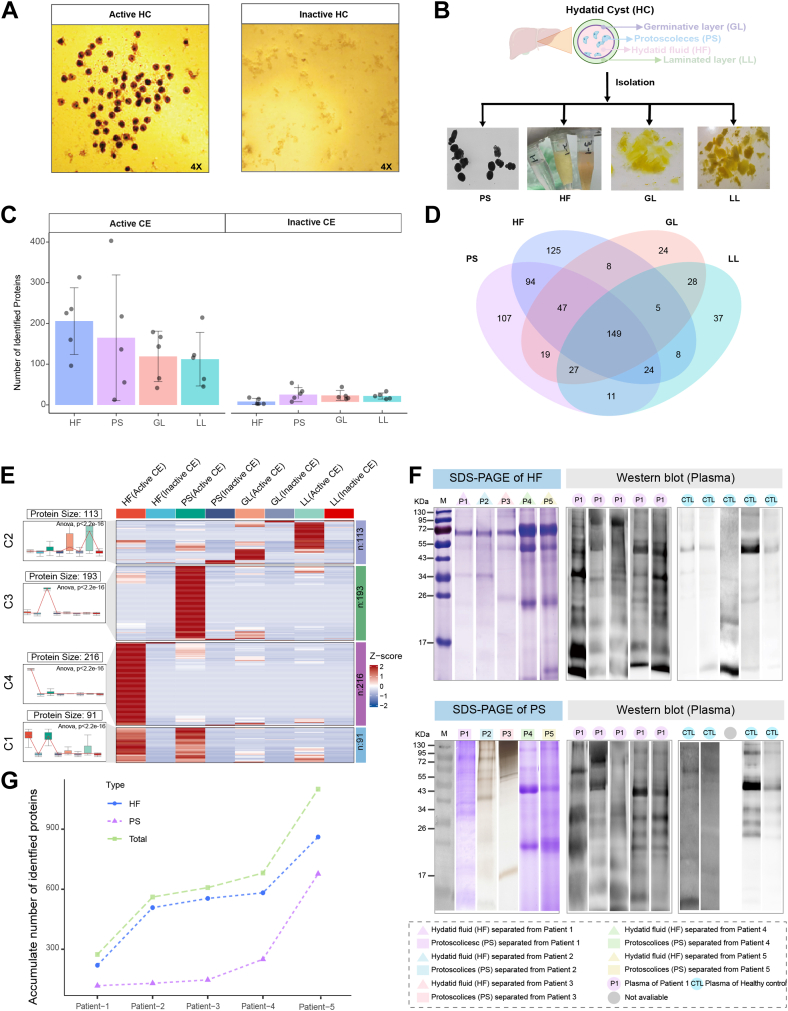
**Profiling of the proteins from the subtissues of HC tissues.***A*, microscopic view of echinococcosis *(left*: active; *right*: inactive). *B*, isolation of HC subtissues from hydatid cyst. *C*, the proteins were identified in the HC subtissues, HF, PS, GL, and LL, which were obtained from five active and five inactive CE patients. *D*, Venn plot of the identified proteins across the HC subtissues. *E*, heatmap of Eg proteins quantified by different subcompartments, HF, PS, GL, and LL from active or inactive CE patients, with median abundance (scaled as *z*-score) from each group. The proteins were clustered by their abundance patterns into different groups. *F*, the typical images of SDS-PAGE and immunoblotting of the HF (*upper*) and PS *(lower*) proteins from five individual CE patients, in which the corresponding CE patient plasma was used as the primary antibodies against the HF or PS proteins. *G*, accumulated number of identified proteins from HF (*blue line*) or PS (*purple line*), whereas the total accumulated proteins from HF and PS are represented by the *orange line*. CE, cystic echinococcosis; Eg, *Echinococcus granulosus*; GL, germinative layer; HC, hydatid cyst; HF, hydatid fluid; LL, laminated layer; PS, protoscoleces.

According to the World Health Organization-Informal Working Group on Echinococcosis definition, CE patients were categorized into active and inactive groups based on viable PS presence. HC tissues from 10 CE patients (five active, five inactive) were surgically resected. Microscopic examination revealed abundant PS in active cases compared with inactive cases (Fig. 1*A*). Individual tissues were dissected into four anatomical subtissues (Fig. 1*B*): PS, HF, GL, and LL. Extracted proteins underwent tryptic digestion and LC–MS/MS analysis. Protein profiling revealed significantly higher Eg proteins in active *versus* inactive HC tissues (Fig. 1*C*). Venn diagram analysis showed that approximately 80% of proteins detected in GL and LL were shared with those in PS and HF (Fig. 1*D*). However, PS and HF exhibited substantially higher protein abundance than GL and LL (Fig. 1*E*). This difference was statistically significant (ANOVA, *p* < 0.01), indicating PS and HF contained the majority of Eg proteins and providing insights into protein distribution across different anatomical structures.

PS and HF proteins from five active CE patients were separated by SDS-PAGE and subjected to immunoblotting using corresponding CE patient plasma or healthy plasma. Five CE patient plasma samples and one pooled healthy plasma were tested against extracted proteins (Supplemental Fig. S1*A*). Typical results (Fig. 1*F*) demonstrated that HF and PS proteins exhibited broad immunoreactivity across wide molecular mass ranges against CE patient plasma, while showing low reactivity against healthy plasma. This confirms specific antibody recognition of antigenic HF and PS proteins (Supplemental Fig. S1*B*). Immunoreactive protein bands were extracted for in-gel tryptic digestion and LC–MS/MS identification. A total of 688 and 859 unique Eg proteins were identified from PS and HF immunoreactive bands, respectively (Fig. 1*G*). After removing redundancies, 1097 Eg proteins were compiled as primary candidates for antigenicity assessment. A pooled healthy-plasma sample was included to distinguish truly parasite-specific immunoreactivity from human background; only bands that were visibly darker than the healthy-pool lane and recognized by at least two individual CE plasma were excised for MS. The immunoblot bands to MS IDs are listed in Supplemental Table S1. This immunoproteomic screening enables antigenicity prediction analysis across distinct HC subtissues.

### Prioritization and Selection of Highly Antigenic Eg Proteins for Recombinant Expression

**Fig. 2:**
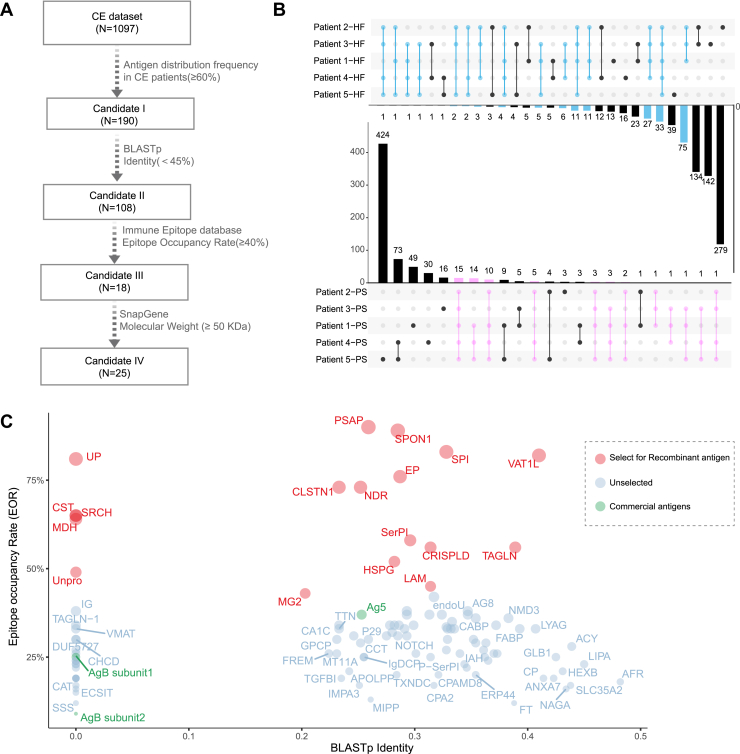
**Selection of the Eg proteins with higher antigenicity for preparation of the corresponding recombinants.***A*, the selection procedure of some Eg proteins with certain criteria. *B*, UpSet plot of the proteins identified in HF and PS subcompartment. The matrix below the bar plot indicates which sets of proteins are represented by each bar. The bar plots on the *left* show the total number of proteins identified in eight patients. *C*, scatterplot of candidate II with BLASTp Identity as *x*-axis and epitope occupancy rate (EOR) as *y*- axis. Eg, *Echinococcus granulosus*; HF, hydatid fluid.

A four-stage bioinformatic filtering process was implemented to identify highly immunogenic Eg proteins suitable for serodiagnosis from 1097 initially identified proteins (Fig. 2*A*). At stage 1, a 60% identification rate cutoff was applied across surgically resected HC samples. The UpSet plot (Fig. 2*B*) demonstrated the distribution of identified Eg proteins in immunoreactive SDS-PAGE bands across five PS and five HF subtissues, with proteins coidentified in more than three subtissues prioritized (red dots). After redundancy removal, 910 proteins were filtered out, yielding 190 high-frequency candidates (candidate I, Supplemental Table S2). At stage 2, BLASTp (30) analysis evaluated sequence similarity between candidate I and human proteins. Although no universal BLASTp identity cutoff is set for protein differentiation, the sequence identity threshold of around 40% to 50% is generally considered to distinguish functionally similar proteins, because below the criteria, function similarity becomes less predictable. Twenty-five proteins showed no human sequence alignments, and an additional 83 candidates were obtained from the remaining 165 proteins after filtering highly similar sequences (>45% identity threshold), generating 108 Eg proteins with low human homology (candidate II). At stage 3, the Immune Epitope Database (31) was employed to predict B-cell epitopes and assess human IgG binding affinities for candidate II proteins. Using epitope occupancy rate (EOR) as the metric for epitope density, and based on commercial hydatid antigen mixture (commercial Egr) benchmarks—antigen B subunits 1 and 2 (0% EOR) and antigen 5 (37% EOR)—a 40% EOR threshold was established. Eighteen Eg proteins exceeded this threshold, forming candidate III (Fig. 2*C*). For the sake of evaluating the protein properties during filtering process of Eg proteins with potential antigenicity as shown in Figure 2*A*, enrichment bioinformatics for protein biochemical properties, such as Gene Ontology enrichment, SignalP 5.0 (32), TMHMM 2.0 (33), and GPI-anchor, was applied to these candidates at each filtration stages (annotated in Supplemental Table S1). As indicated in Supplemental Fig. S2, at each filtration stage, the candidates with secretory and membrane properties were increased compared with those in the original 1097 Eg proteins. For instance, over 60% (11/18) of candidate III were predicted as secretory or membrane proteins, whereas the ratio in the original 1097 Eg proteins was about 31%. Since protein antigenicity is assumed to be tightly associated with its secretory or hydrophobicity property, the enrichment analysis is likely to strengthen the rationality of the filtration process for Eg proteins with potential antigenicity.

At stage 4, we first tallied the 18 full-length sequences retained after bioinformatic filtering (candidate III). Because *E. coli* expression efficiency drops sharply above ∼70 kDa, we re-examined the four largest proteins: cystatin (CST, 82 kDa), spondin-1 (SPON1, 105 kDa), proactivator polypeptide (PSAP, 94 kDa), and murinoglobulin-2 (MG2, 168 kDa). Each was computationally split using SnapGene into two or three nonoverlapping, domain-respecting fragments, yielding nine extra sequences (listed in Supplementary Table S3). Consequently, 18 intact ORFs plus nine truncated fragments gave 25 distinct constructs (termed candidate IV), which proceeded to cloning and expression. All genes were successfully cloned into pET-30a (+) vectors, with 18 recombinants expressed in *E. coli*.

### Evaluation of Plasma Immunoreactivity Against Eg Recombinant Antigens

**Fig. 3:**
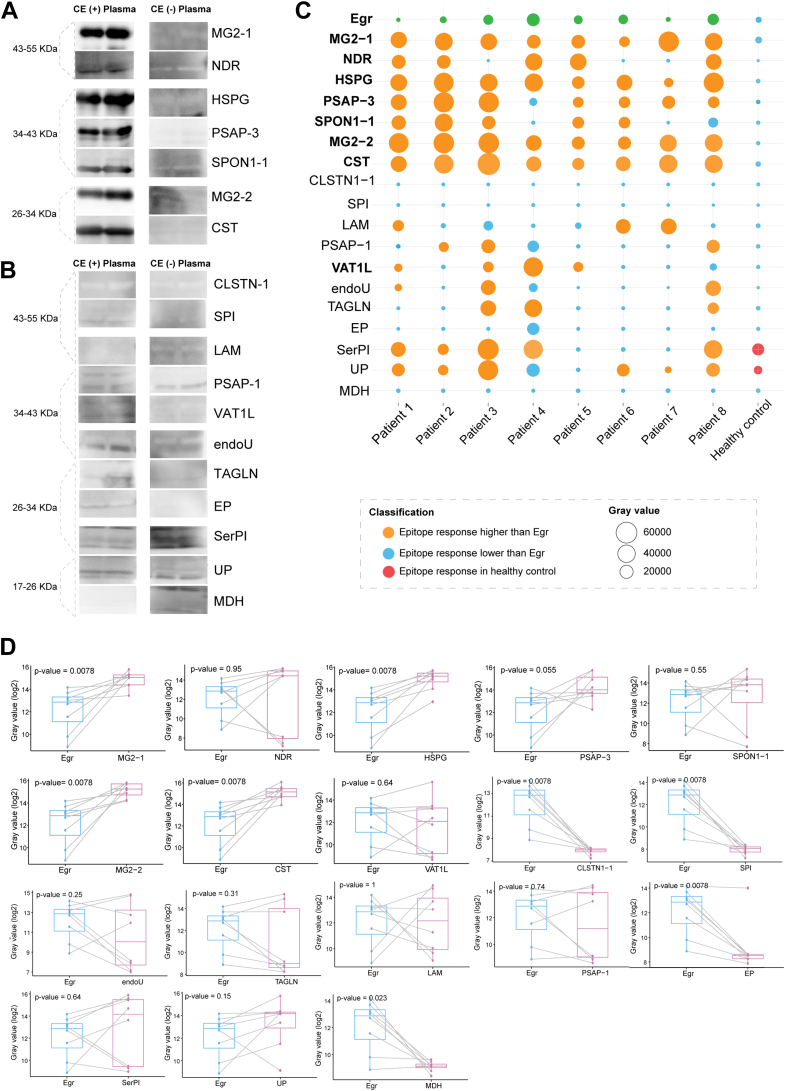
**Immunoreactivity evaluation of the Eg recombinants.***A*, immunoblotting images of the seven Eg recombinants with positive immunoreactivities, in which CE-positive (+) plasma indicates a CE plasma as primary antibody and CE-negative (−) plasma denotes a pooled healthy plasma as primary antibody. *B*, immunoblotting images of the 11 Eg recombinants with negative immunoreactivities, in which CE positive (+) represents the same meaning as above. *C*, bubble plot of the immune reactivity of different Eg recombinants examined by the eight different CE plasmas, in which dot size represents *gray values* of immunoreactive intensities. In the plot, the pooled health plasma is set as a negative control, whereas commercial Egr is defined as a positive control. *Orange bubbles* represent an Eg recombinant with immunoreactivity higher than that of commercial Egr and no crossreactivity against health plasma; *blue bubbles* stand for an Eg recombinant with immunoreactivity lower than that of commercial Egr and no crossreactivity against health plasma; and *red bubbles* denote an Eg recombinant with immunoreactivity against health plasma. *D*, paired differential test of *gray values* for different Eg recombinant proteins using eight CE plasma samples as primary antibody, with commercial Egr as control. CE, cystic echinococcosis; Eg, *Echinococcus granulosus*.

Immunoblotting analysis using plasma from eight CE patients revealed clear recognition of Eg recombinants, while showing no significant response to healthy plasma (Fig. 3, *A* and *B*). ImageJ analysis quantified immunoreactivity patterns across all 18 recombinants (Fig. 3*C*, Supplemental Table S4). Eight Eg recombinants met the stringent selection criteria: (1) no reactivity against healthy plasma and (2) immunoreactivity equal to or exceeding commercial Egr in >50% of CE patient samples. The selected candidates included CST, heparan sulfate proteoglycan (HSPG), MG2-1 and MG2-2 (MG-2 truncates), PSAP-3 (proactivator polypeptide truncate), nuclear Dbf2-related kinase (NDR), SPON1-1 (SPON1 truncate), and vesicle amine transport protein 1 homolog-like (VAT1L).

Paired difference analysis of gray values from eight CE patient samples demonstrated that the selected antigens either exhibited significantly higher immunogenicity than commercial Egr (paired *t* test, *p* < 0.05) or displayed comparable performance (Fig. 3*D*). Nonselected antigens consistently showed lower immunogenicity relative to commercial Egr, confirming the robustness of the selection criteria and the strong immunogenic potential of the chosen candidates for further investigation.

### Large-Scale ELISA Validation and Performance Assessment

**Fig. 4:**
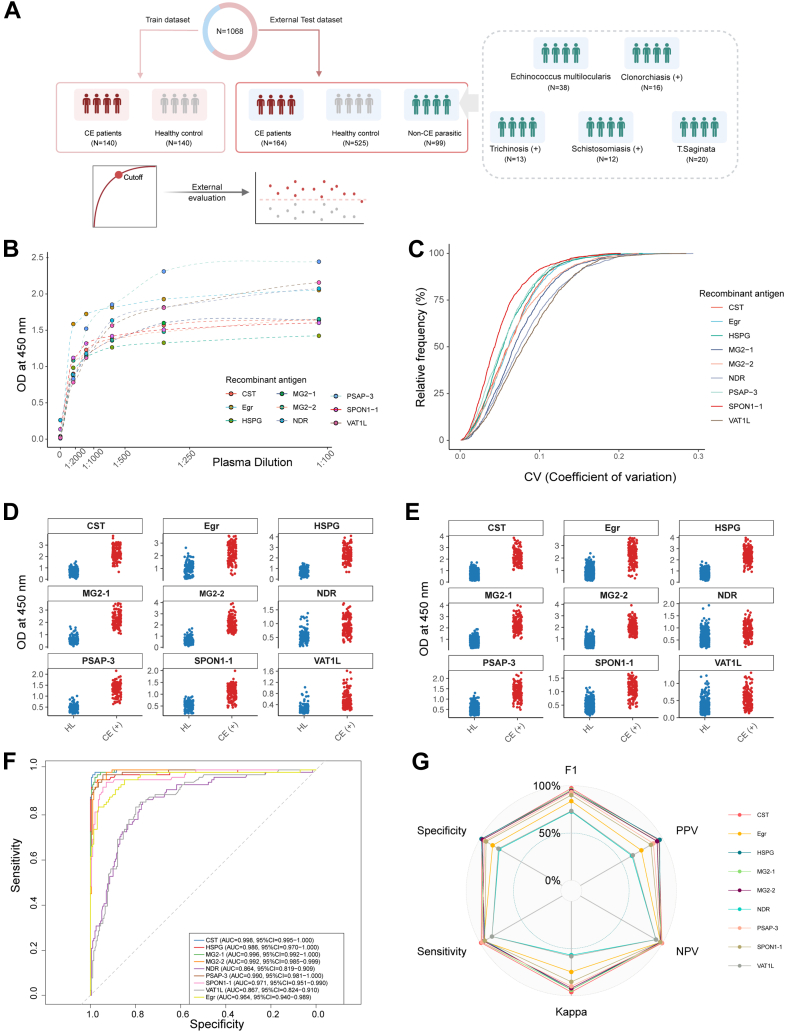
**Immunoreactivity evaluation toward the eight Eg against the plasma sample sets collected from CE and health at a large scale.***A*, sample set generation for better evaluation toward the Eg recombinants in large plasma samples. *B*, titration curves for the eight prioritized Eg recombinants and commercial Egr, a commercial antigen. *C*, cumulative coefficients of variation (CVs) of the ELISA measurements for individual Eg recombinants. *D*, scatter plots of ELISA results using our novel eight recombinant antigens and the commercial Egr as reference antigens. The plasma samples used were from healthy controls (HL) and CE (+). The scatter plot indicates the immune level of absorption at an absorbance of 450 nm in the train dataset. *E*, scatter plots of ELISA results using eight recombinant antigens and the commercial Egr as reference antigens. The plasma samples used were from HL and CE (+). The scatter plot indicates the immune level of absorption at an absorbance of 450 nm in the external test dataset. *F*, AUROC curves of the individual Eg recombinants in sample set 2. *G*, performance evaluation toward the nine Eg proteins with sensitivity, specificity, accuracy, PPV, NPV and F1 score, respectively. AUROC, area under the receiver operating characteristic curve; CE, cystic echinococcosis; Eg, *Echinococcus granulosus*; NPV, negative predictive value; PPV, positive predictive value.

Nine antigens, including the eight selected candidates and commercial Egr as control, were evaluated using 969 plasma samples randomly divided into two sets (Supplemental Fig. S3). Sample set 1 comprised 140 CE-positive patients (confirmed by ultrasound) and 140 healthy controls (HLs) as the training dataset to establish ELISA absorbance value criteria, whereas sample set 2 contained 164 CE-positive patients and 525 HLs for independent external validation (Fig. 4*A*). Prior to ELISA analysis, optimal antigen concentrations and plasma dilution factors were determined through checkerboard titration (Supplemental Table S5), with absorption at absorbance at 450 nm plotted against various plasma dilution factors (Fig. 4*B*). All antigens underwent large-scale ELISA screening with triplicate measurements, achieving cumulative coefficients of variation below 15% for technical replicates (Fig. 4*C*).

Significant differences in absorbance values between CE-positive subjects and HLs were observed in both training (Fig. 4*D*, Supplemental Table S6) and external test datasets (Fig. 4*E*, Supplemental Table S7). In the training dataset, seven Eg antigens (CST, commercial Egr, HSPG, MG2-1, MG2-2, PSAP-3, and SPON1-1) demonstrated excellent performance with AUROC values exceeding 0.95, whereas two antigens (NDR and VAT1L) showed robust performance with AUROC values around 0.85 (Supplemental Table S8). Cutoff values were determined using the Youden index to optimize sensitivities, specificities, positive predictive values, negative predictive values, and Kappa coefficients. External validation using sample set 2 revealed that five Eg recombinants (CST, HSPG, MG2-1, MG2-2, PSAP-3, and SPON1-1) exhibited superior diagnostic capabilities with sensitivities ranging from 91.26% to 99.09% and specificities from 95% to 97% (Fig. 4, *F* and *G*, Supplemental Table S9). These recombinants significantly outperformed commercial Egr, which showed 73.89% sensitivity and 92.05% specificity. The remaining three recombinants (SPON1-1, VAT1L, and NDR) demonstrated performance comparable to commercial Egr.

**Fig. 5:**
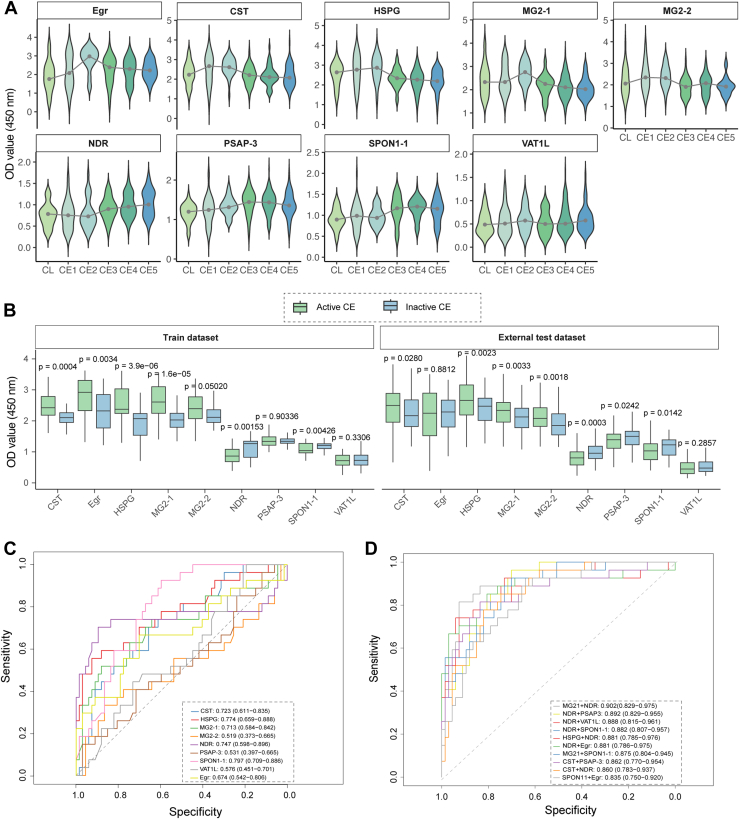
**Discriminator construction to distinguish different CE stages based on the combination of novel Eg recombinants.***A*, distribution of absorbance at 450 nm values for different CE stages of each antigen. *B*, differential analysis of absorbance at 450 nm values for each antigen between active and inactive CE group, in the train dataset and external test dataset. *C*, AUROC of single antigen for distinguishing active and inactive CE in the external test dataset. *D*, AUROC of two combined antigens for distinguishing active and inactive CE in the external test dataset, with only top10 combinations were plotted. AUROC, area under the receiver operating characteristic curve; CE, cystic echinococcosis; Eg, *Echinococcus granulosus*.

CE patients were stratified by cyst type into groups: cystic lesion (CL) (n = 29), CE1 (n = 46), CE2 (n = 32), CE3 (n = 41), CE4 (n = 99), and CE5 (n = 57). Immune response levels against Egr, CST, HSPG, MG2-1, and MG2-2 were significantly higher in active CE stages (CE1, CE2) compared with inactive stages (CE3, CE4, and CE5). Conversely, NDR, PSAP-3, and SPON1-1 showed higher responses in inactive CE stages, whereas VAT1L demonstrated no significant differences across CE stages (Fig. 5*A*). For discriminating between active (CE1, CE2) and inactive (CE4, CE5) CE forms, individual antigens showed limited performance with AUROC values ranging from 51.9% to 79.7% (Fig. 5, *B* and *C*). However, combinations of two Eg proteins achieved significantly improved discrimination with median AUROC values exceeding 0.90. Using SVM modeling, the optimal combination of MG2-1 and NDR achieved an AUROC of 0.902 (95% confidence interval: 0.829–0.975) in external sample set 2 (Fig. 5*D*).

### Cross-reactivity in Nonechinococcus Parasitic Infections for the Serological Diagnosis of CE

To evaluate the discriminative capacity of the novel Eg recombinant panels, we assessed their ability to distinguish between different echinococcosis types, CE and alveolar echinococcosis (AE, N = 38), and tested crossreactivity with other parasitic infections. The immune response analysis revealed that most antigens (CST, commercial Egr, HSPG, MG2-2, NDR, PSAP-3, SPON1-1, and VAT1L) showed comparable absorbance values in both CE and AE plasma samples, with only MG2-1 demonstrating higher absorbance values in CE patients (Supplemental Fig. S4*A*). Given the high genomic sequence homology (>96%) between parasites (Eg, Em) causing AE and CE, the similar immune responses may explain this moderate discrimination capability. Consequently, AE and CE patients were combined as cyst (+) for subsequent analyses.

Cross-reactivity evaluation was performed using plasma samples from patients with confirmed infections by various cestodes, including *Clonorchiasis*, *Schistosomiasis*, *T. saginata*, and *Trichinosis*, all sourced from the same geographic region. Testing of the eight novel Eg recombinants and commercial Egr revealed that each antigen exhibited significantly higher absorbance values in ultrasound cyst (+) patients compared with patients with other parasitic diseases (Supplemental Fig. S4*B*). This pattern indicates minimal crossreactivity and suggests that these antigens serve as reliable biomarkers specifically recognized by antibodies in cyst (+) patient plasma.

**Fig. 6:**
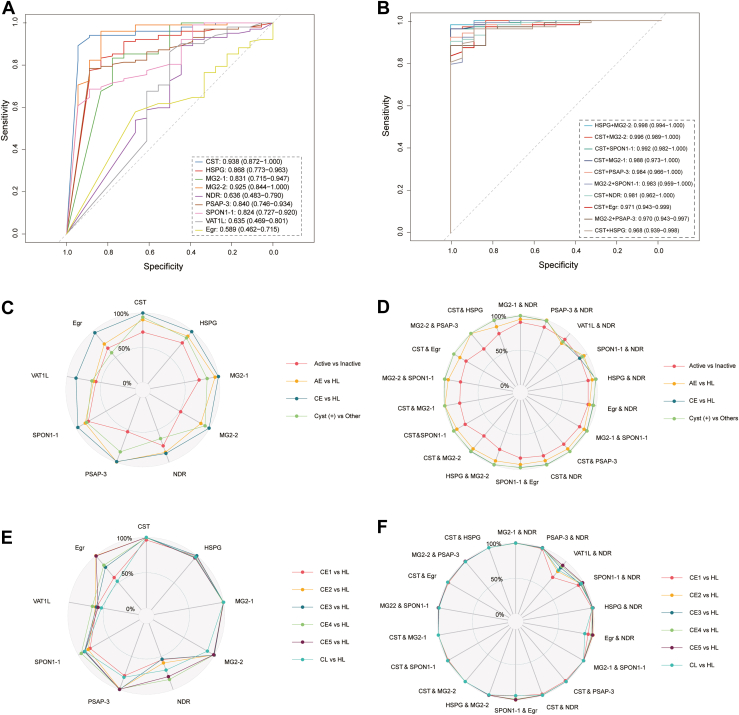
**Discriminator construction to evaluate the crossreactivity in non-Echinococcus parasitic infections for the serological diagnosis of cystic echinococcosis (CE).***A*, AUROC of single antigen for distinguishing cyst (+) and other non-Echinococcus parasitic infection groups in the external test dataset. *B*, AUROC of two combined antigens for distinguishing cyst (+) and other non-Echinococcus parasitic infection groups in the external test dataset, with only top10 combinations plotted. *C*, AUROC of various echinococcosis antigens in distinguishing different groups: CE *versus* healthy controls (HL), AE (alveolar echinococcosis) *versus* HL, cyst (+) *versus* HL, active *versus* inactive. *D*, AUROC based on the combinations of two antigens, such as CST and HSPG, MG2-1 and NDR, PSAP-3 and NDR, VAT1L and NDR, HSPG and NDR, AE *versus* HL, CE *versus* HL, Egr and NDR, and cyst (+) *versus* others. *E*, AUROC of various echinococcosis antigens in distinguishing between CE stages (CE1–CE5) and HL, as well as cystic lesions (CL) *versus* HL. *F*, AUROC based on the combinations of two antigens, such as CST and HSPG, MG2-1 and NDR, PSAP-3 and NDR, VAT1L and NDR, HSPG and NDR in distinguishing between CE stages (CE1–CE5) and HL, as well as CL *versus* HL. AUROC, area under the receiver operating characteristic curve; CST, cystatin; HSPG, heparan sulfate proteoglycan; MG2, murinoglobulin-2; NDR, nuclear Dbf2-related kinase; PSAP, proactivator polypeptide; VAT1L, vesicle amine transport protein 1 homolog-like.

Single antigen analysis demonstrated robust discriminative performance for several candidates. CST, MG2-2, and HSPG showed superior performance with AUROC values of 0.938, 0.925, and 0.868, respectively, in the external dataset, all exceeding the commercial Egr benchmark (Fig. 6*A*). When evaluating two-antigen combinations for discriminating cyst (+) from non-Echinococcus parasitic infections, most combinations achieved AUROC values above 0.96. The optimal combination of HSPG and MG2-2, using an SVM model, yielded an exceptional AUROC of 0.998 (95% confidence interval: 0.994–1.000) in external sample set 2 (Fig. 6*B*).

Radar plot analysis provided a comprehensive visualization of antigen performance across multiple discrimination tasks. Individual antigen performance was assessed for CE (+) *versus* HL, AE (+) *versus* HL, cyst (+) *versus* other parasitic diseases, and active *versus* inactive CE (+) (Fig. 6*C*). Two-antigen combinations showed enhanced performance across these categories (Fig. 6*D*). Additional analysis focused on differentiating CE stages (CE1–CE5) and CLs from HLs, demonstrating the diagnostic potential of both individual antigens (Fig. 6*E*) and their combinations (Fig. 6*F*) across various disease stages. These results demonstrate that the selected Eg recombinants exhibit strong diagnostic potential with minimal crossreactivity, supporting their utility as reliable biomarkers for echinococcosis screening and staging.

## Discussion

Current serological testing for CE shows limited effectiveness because of insufficient availability of Eg antigens with robust diagnostic performance. Our immunoproteomic workflow addresses this critical gap by identifying and validating CE-specific antigens for clinical application. Our approach incorporates several key strategies that facilitate antigen discovery. First, sampling of HC tissues from patients at different disease stages, combined with LC–MS/MS–based protein profiling, enabled identification of tissue-specific antigen expression patterns. PS and HF from active cysts contained diverse antigenic proteins, consistent with their role in active infection. Second, crossvalidation using multiple CE patient plasma samples ensured robust antigen selection. This dual verification approach—testing both native tissue proteins and recombinant antigens against diverse patient sera—minimized false-positive candidates and enhanced clinical relevance. Third, high-resolution MS identified 1097 Eg proteins, representing a substantial increase over previous studies (20–280 proteins). This expanded protein database facilitated more comprehensive antigen screening and improved candidate selection.

The Eg proteins with higher antigenicity found in this study emerge the intriguing objects for clinical application onto the CE serological test. CST, a cysteine-type protease inhibitor with Kunitz and Kazal domains, was identified in the HF compartment of Eg and *Echinococcus ortleppi* pulmonary bovine cysts (24), whereas our study further confirmed the CST presence in HC tissues, PS and HF. Moreover, for the first time, the strong immunoreactivity of the CST recombinant against the CE plasma was corroborated in a large sample set of patient plasma. HSPGs are glycoproteins characterized by one or more covalently attached heparan sulfate chains. They are found on the cell surface and in the extracellular matrix, exerting significant influence on growth factor activity, cell adhesion, and tissue structure (34). The HSPGs secreted from parasites promote microbial infection. Ahn *et al* (35). identified HSPG as an abundant protein in Em and Eg and predicted HSPG as a potential hydatid antigen. Comprehensive validation with a larger sample set in this study provides robust evidence for HSPG’s diagnostic utility for CE. MG-2, an Eg protein with 637 amino acids, has previously been reported only as a putative secretory protein based upon analysis of ORFs (36). The results in this study reveal MG2's robust antigenic properties. The identified MGs are divided into two recombinants, MG2-1 and MG2-2, for ELISA validation, whereas the two recombinants are well qualified in specific and sensitive recognition to the CE plasma. PSAP is a small protein associated with cell differentiation in adult worms (Adult), oncospheres, PS, and HC membrane (Cyst) of Eg (37).

In the near future, acceleration of clinical application for these Eg antigens potentially sensitive to the CE patient plasma is a working direction in our laboratory. Briefly, three aspects of the application of new Eg biomarkers are mainly considered. (1) A panel with minimal Eg antigens in ELISA should be carefully assessed and designed based on the eight Eg antigens described above. For instance, the ELISA tests with single antigens suggest CST, HSPG, and MG2-2 as front-line candidates (AUROC above 0.92, sensitivity/specificity both above 0.9), which might be translated into the lateral-flow platform as a prescreen. While a dual-antigen ELISA with a combination of HSPG + MG2-2 can improve AUROC to 0.998. (2) In further clinical deployment, we will replace patient-derived QC with commercial-plasma standards spiked at three antigen levels and convert absorbances to fixed “ELISA units.” (3) Generation of stable Eg antigens at manufacturing scale is a necessary step to make the conventional ELISA kits for clinical tests. Although the strategy of truncated recombinants was engaged in this study, expression of recombinant using *E. coli* system would not be the best choice. Eg peptide synthesis based on the assistance of artificial intelligence is likely to be an important approach. (4) Current examination upon 600 CE patients is far from satisfactory for clinical diagnosis using a new kit of Eg antigen. Therefore, expansion of the collaboration network, especially working with the hospitals in hyperendemic regions, would greatly help robust control and quantitative estimation of the ELISA kit with new Eg antigens.

In this study, we developed a screening method to identify individuals at risk of infection, but it does not differentiate between current and past infections. It is important to acknowledge the limitations of our study. Although the individual Eg recombinants and their combinations could achieve satisfactory AUROC values, several factors leading to false positives should be fully acknowledged. First, the cohort with unbalanced samples is likely to introduce statistical bias, with only 61 non-CE parasitic samples *versus* 303 CE cases, which might result in a potentially overoptimistic AUROC. Second, this study contained only 29 ultrasound-negative (CL) patients available, who were lack of long follow-up. Large ultrasound-negative samples with long follow-up are necessary for further validating the negative prediction for progression of active CE1 to CE2 stages. Third, the absence of a true ultrasound-negative sample that is epidemiologically exposed to a special environment limits a correct estimation toward the panel's negative predictive value for incident infection. The crossreactivity of the identified antigens was tested in a limited number of samples from patients with various parasitic diseases. Future clinical applications will require validation with larger sample sets from multiple parasitic diseases to ensure the antigens' specificity and reliability. In addition, the clinical application of these antigens will necessitate the establishment of QC materials, standardization, and calibration, as well as clinical trials to ensure their accuracy and consistency across different centers. Overall, the results achieved from the innovative workflow widen our view of the Eg proteins and uncover the promising candidate potential in the serological test of CE.

## Data Availability

The proteomics data generated from this study have been deposited in the iProX Integrated Proteome resources (https://www.iprox.cn/page/home.html; accession number: IPX0009327000).

## Supplemental Data

This article contains supplemental data.

## Conflict of Interest

The authors declare no competing interests.
