# Supplementary material for: Patient Plasma–Based Immunoproteomics Reveals Novel Echinococcus granulosus Antigens for Diagnosis of Cystic Echinococcosis
Source: Mol Cell Proteomics. 2025 Dec 8;25(1):101485. doi: 10.1016/j.mcpro.2025.101485 (PMC12809694; doi:10.1016/j.mcpro.2025.101485)
Supplement: Supplemental information [file mmc10.docx]

**Supplemental information**

**Patient plasma-based immunoproteomics reveals novel *Echinococcus granulosus* antigens for diagnosis of cystic echinococcosis**

*Congmin Zhang^1^#, Quzhen Gongsang^2^#, Wangmu Danzeng^1^, Xi Gao^1^, Yuxin Li^1^, Yanping Zhao^3^, Hongkai Xu^1^, Cong Wang^1^, Ting Zhang^4^, Muxin Chen^4^, Yijun Tang^5^, Jiawei Liu^1^, Jin Zi^1^, Liang Lin^1^, Guixue Hou^1^*, Siqi Liu^1^**

1. BGI Genomics, Shenzhen 518083, China
2. NHC Key Laboratory of Echinococcosis Prevention and Control, Tibet Center for Disease Control and Prevention, Lhasa 850000, China
3. BGI Research, Chongqing 401329, China
4. National Institute of Parasitic Diseases, Chinese Center for Disease Control and Prevention, Chinese Center for Tropical Diseases Research, Shanghai, 200025, China
5. Shenzhen Center for Disease Control and Prevention, National Institute of Parasitic Diseases, Shenzhen, Guangdong 518073, China

#These authors contributed equally to this work.

*These authors are co-corresponding authors.

**Supplemental Data**

Supplemental Table 1: Detailed information of identified Eg proteins.xlsx.

Supplemental Table 2: Detailed information of Candidate I.xlsx.

Supplemental Table 3: Detailed information of 25 recombinant proteins.xlsx.

Supplemental Table 4. Gray values of immunoblotting for Eg recombinant proteins with plasma of CE or healthy samples as primary antibody.xlsx

Supplemental Table 5. Optimal recombinant antigen concentration and plasma dilution factor used for ELISA.xlsx

Supplemental Table 6. Clinical information and ELISA quantification results of the train data set.xlsx

Supplemental Table 7. Clinical information ELISA quantification results of the test data set and cross-reactivity evaluation.xlsx

Supplemental Table 8. Performance of single antigen in the diagnosis of CE (+) and healthy control in the train data set.xlsx

Supplemental Table 9. Performance of single antigen in the diagnosis of CE (+) and healthy control in the test data set.xlsx

Supplemental Figure 1. Design of antigen cross-validation with immunoblotting.

Supplemental Figure 2. Protein properties analysis for 1,100 Eg proteins.

Supplemental Figure 3. Flowchart of the clinical subjects for serological diagnosis of cystic echinococcosis.

Supplemental Figure 4. Discriminator construction to evaluate the cross-reactivity in non-Echinococcus parasitic infections for the serological diagnosis of cystic echinococcosis.


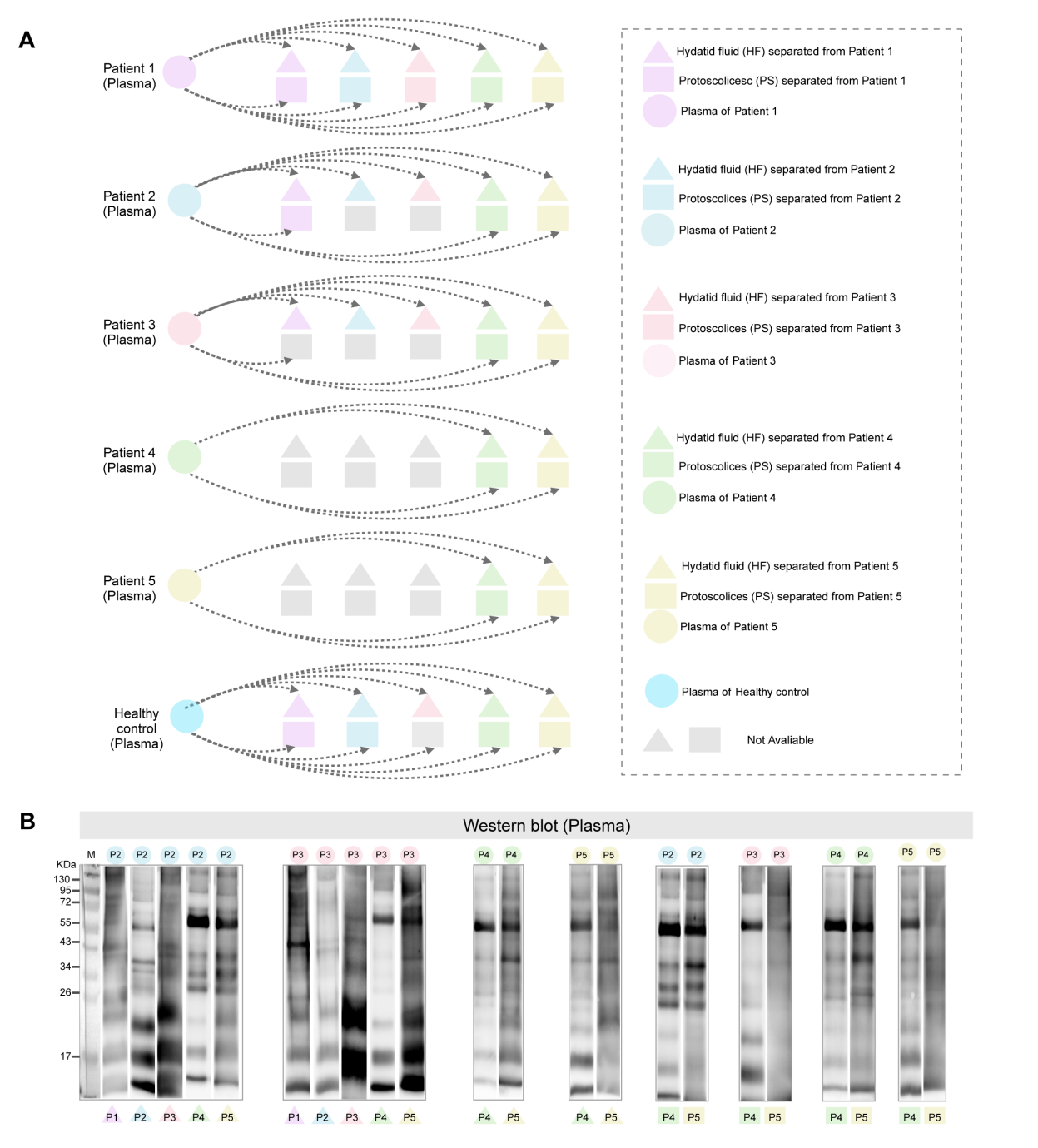


**Supplemental Figure 1.** A) Five CE patients and one healthy control subject were selected for analysis and annotated with different colors. Triangles represent hydatid fluid separated from patients, rectangles represent protoscoleces (PS) separated from patients, and circles represent plasma from patients. If there were no enough proteins for immunoblotting, the color was grey. B) The typical images of SDS-PAGE and immunoblotting of the HF (Triangles) or protoscoleces (PS) (rectangles) proteins from 5 individual CE patients, in which the corresponding CE patient plasma as primary antibodies against the HF or protoscoleces (PS) proteins.


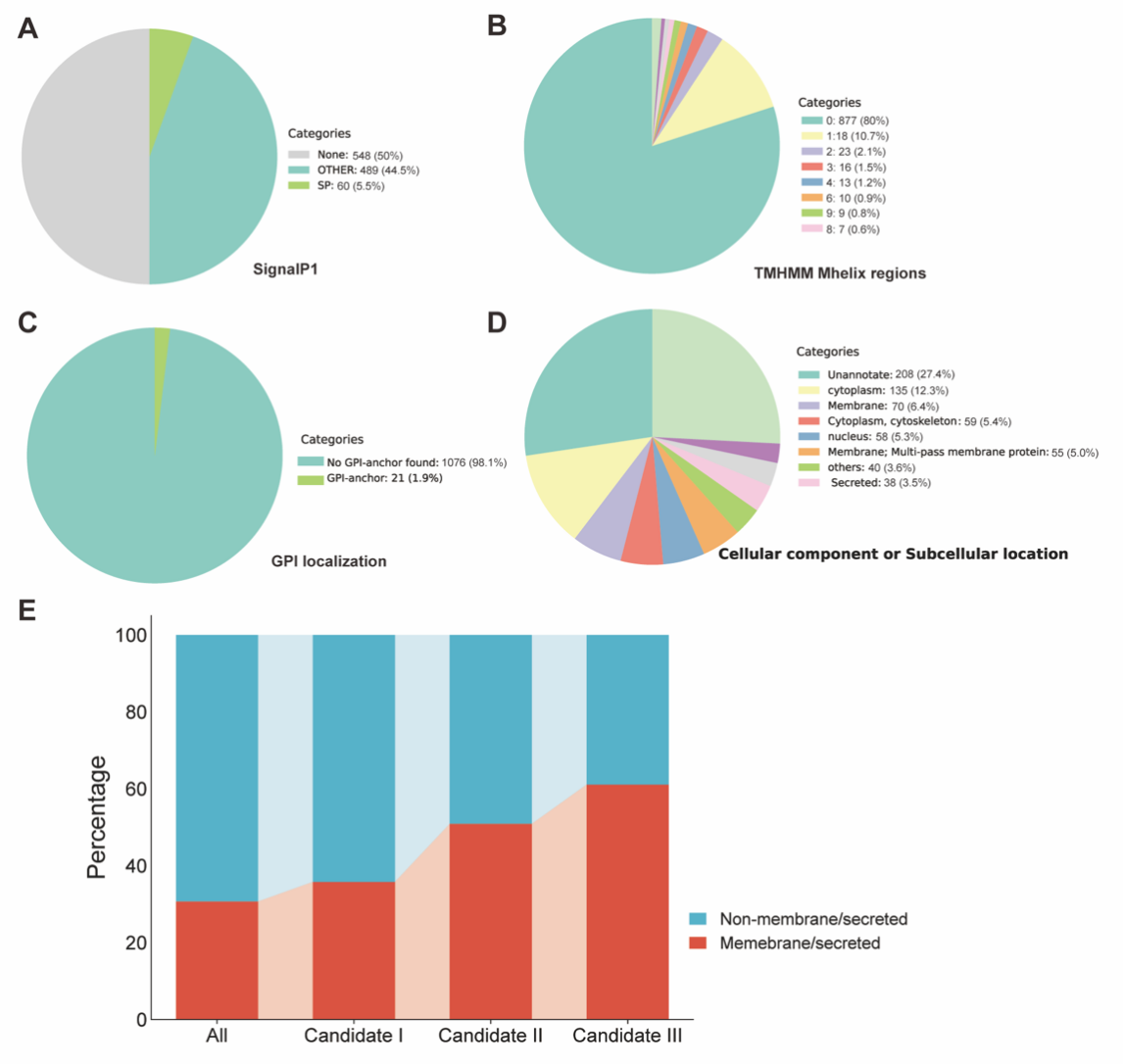


**Supplemental Figure 2. Protein properties analysis for 1,097 Eg proteins.** Global distribution of 1,100 proteins predicted by SignalP (A), TMHMM (B), GPI‑anchor (C) or annotated as extracellular GO terms (D) in the uniprot database. (E) Percentage distribution for proteins in candidate lists predicted with secreted or membrane properties.

**
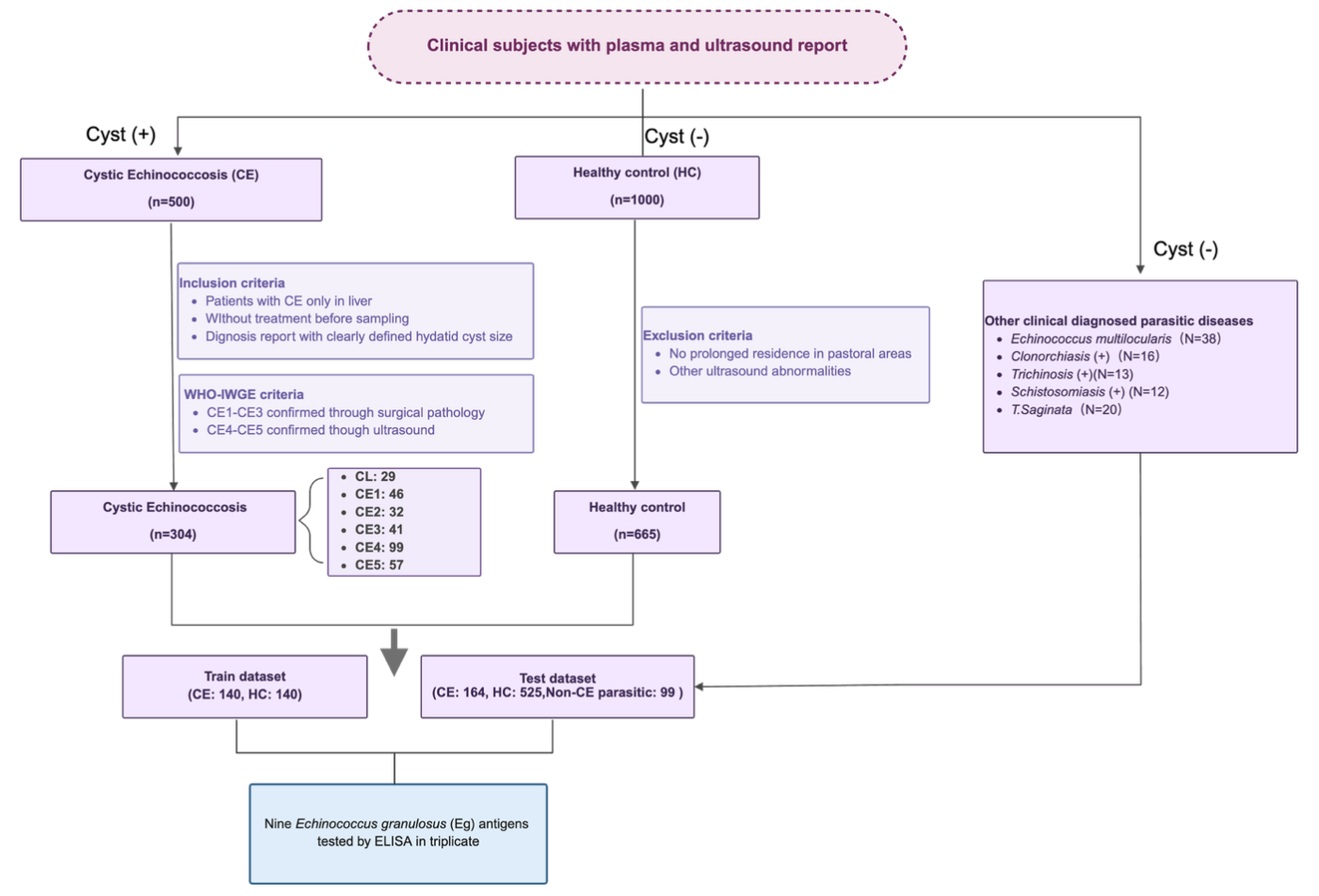
**

**Supplemental Figure 3. Flowchart of the clinical subjects for serological diagnosis of cystic echinococcosis.** Plasma samples were collected from 304 cystic echinococcosis (CE) patients with liver-localized, untreated lesions (WHO-IWGE stages CE1–CE5) and 665 healthy controls (HC). Individuals with other parasitic infections (E. multilocularis, clonorchiasis, trichinellosis, schistosomiasis, Taenia saginata) were selected for cross-reactivity evaluation. For serological profiling, nine Echinococcus granulosus (Eg) antigens were screened by ELISA in triplicate; the final data set comprised a training subset (CE = 140, HC = 140) and an independent test subset (CE = 164, HC = 525, non-CE parasitic diseases = 99).


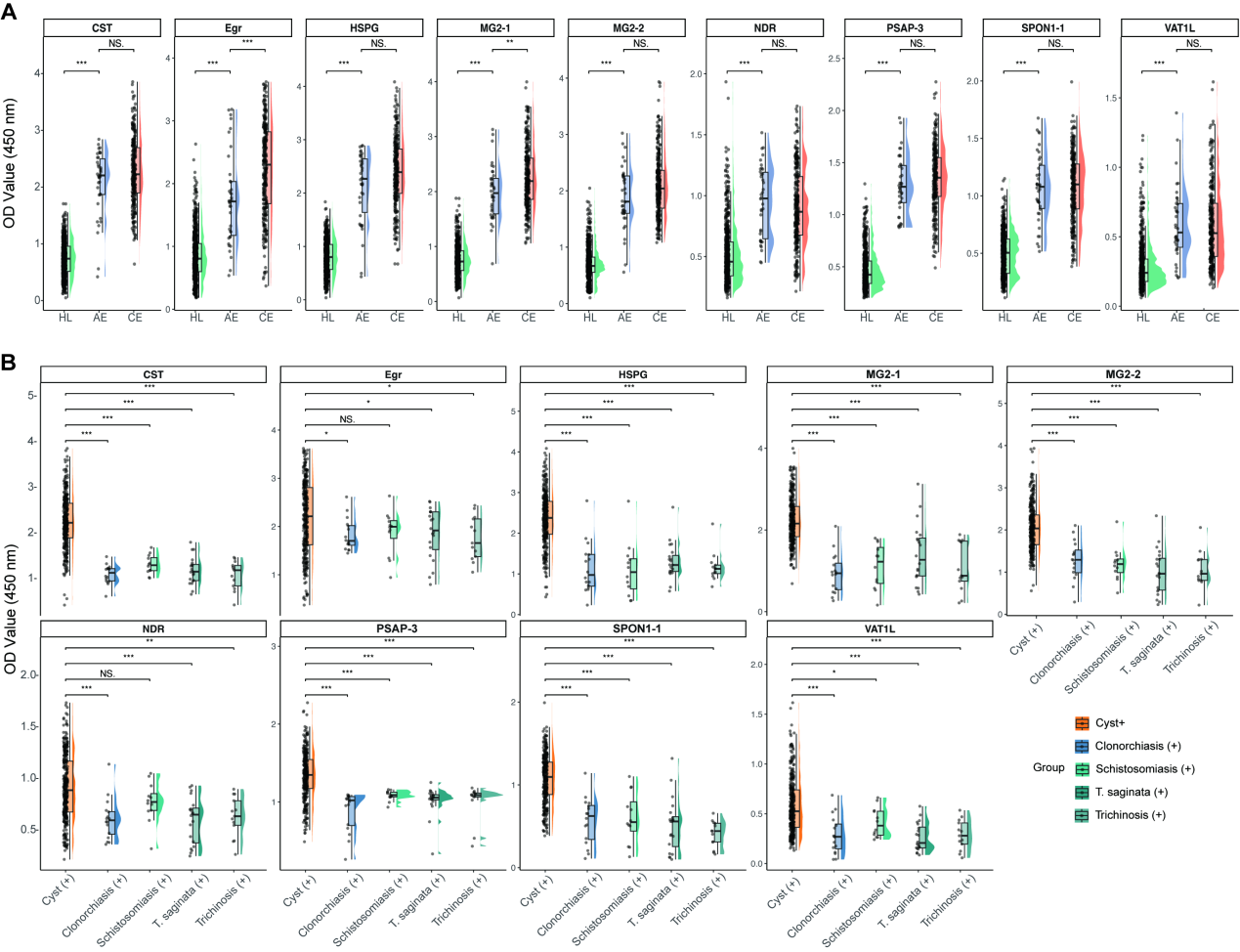


**Supplemental Figure 4.** A) Distribution of the OD 450nm values for each antigen in AE, CE and HL. B) Differential analysis of OD 450 nm values for each antigen between cyst (+) and non-Echinococcus parasitic infection groups, including *Clonorchiasis, Schistosomiasis, T. saginata* and *Trichinosis*.
